# Virtual screenings of the bioactive constituents of tea, prickly chaff, catechu, lemon, black pepper, and synthetic compounds with the main protease (Mpro) and human angiotensin-converting enzyme 2 (ACE 2) of SARS-CoV-2

**DOI:** 10.1186/s43094-021-00275-7

**Published:** 2021-06-15

**Authors:** Nazim Uddin Emon, Md. Munsur Alam, Irin Akter, Saima Akhter, Anjuman Ara Sneha, Md. Irtiza, Marufa Afroj, Arifa Munni, Masruba Hossen Chowdhury, Summiya Hossain

**Affiliations:** 1 Department of Pharmacy, International Islamic University Chittagong, Chittagong, 4318 Bangladesh; 2 Department of Public Health, School of Science and Technology, Bangladesh Open University, Gazipur, 1705 Bangladesh

**Keywords:** SARS-CoV-2, Theaflavin, Kaempferol, Drug targets, Molecular docking

## Abstract

**Background:**

COVID-19 has mutation capability, and there are no specific drug therapies that are available to fight or inhibit the proteins of this virus. The present study aims to investigate the binding affinity of the bioactive and synthetic compounds with the main protease (Mpro) enzymes and angiotensin-converting enzyme 2 (ACE 2) by computational approach. PASS prediction, pharmacokinetics, and toxicological properties prediction studies were performed through the Google PASS prediction and Swiss ADME/T website. Besides, molecular docking studies were accomplished by BIOVIA Discovery Studio 2020, UCSF Chimera, and PyRx autodock vina.

**Results:**

The docking scores were inferred and the selected compounds showed results varying from −3.2 to −9.8 (kcal/mol). Theaflavin scored the highest docking score to the 5REB, 6VW1, and 1R42 enzymes and showed the binding affinity as −6.3 kcal/mol, −9.8 kcal/mol, and −8.6 kcal/mol, respectively. Again, kaempferol showed the best binding affinity to the 7BQY (−7.1 kcal/mol) and 6Y2FB (−6.6 kcal/mol) enzymes. All the chemical constituents showed better probability in action in pass prediction analysis. Besides, no ligands (except theaflavin) have any conflict with Lipinski’s rules of five, which authorized the drug probability of these ligands.

**Conclusion:**

Therefore, the selected compounds could be considered a potential herbal treatment source against SARS-CoV-2.

## Background

According to the WHO (World Health Organization), SARS-CoV-2 is now a pandemic crisis. It first appeared in the Wuhan province of China on 31 December 2019, and spread rapidly in the different parts of the world. Till 30 April 2021, there are 151,837,341 confirmed cases of 2019-nCoV infection, with 3,188,507 deaths found around the globe. The causative agent was identified from throat swab samples conducted by the Chinese Center for Disease Control and Prevention (CCDC) on 7 January 2020, and was subsequently named severe acute respiratory syndrome coronavirus-2 (SARS-CoV-2). The disease was named COVID-19 by the WHO [1]. Most SARS-CoV-2 infected patients are seen with symptoms such as dry cough, sore throat, and fever. The majority of cases have spontaneously resolved. However, some have developed various fatal complications, including organ failure, septic shock, pulmonary edema, severe pneumonia, and acute respiratory distress syndrome (ARDS) [2]. WHO declared the Chinese outbreak of COVID-19 to be a public health emergency of international concern, posing a high risk to countries with vulnerable health systems on 30 January 2020. The emergency committee has stated that the spread of COVID-19 may be interrupted by early detection, isolation, prompt treatment, and the implementation of a robust system to trace contacts [3].

Coronaviruses belong to the Coronaviridae family in the Nidovirales order. Corona contains crown-like spikes on the outer surface of the virus; thus, it was named coronavirus. Coronaviruses are minute in size (65-125 nm in diameter) and contain a single-stranded RNA as a nucleic material, varying in length from 26 to 32 kbs. The subgroups of the coronaviruses family are alpha (α), beta (β), gamma (γ), and delta (δ) coronavirus [4]. SARS-CoV-2 fits the beta Coronavirus group [5]. COVID-19 (SARS-CoV-2) is composed of a spike protein (S), a membrane glycoprotein (M), a hemagglutinin-esterase dimer (HE), an envelope protein (E), a nucleocapsid protein (N), and an RNA. Spike glycoproteins are composed of two subunits (S1 and S2). Homotrimers of S proteins compose the spikes on the viral surface, guiding the link to host receptors [5, 6]. Nucleocapsid protein (N) binds in vitro to RNA and is heavily phosphorylated. N protein binds with the viral genome in a bead on a string type conformation, and E protein is found in small quantities within the virus, whereas the most abundant structural protein is the M protein. This M protein contains no signal sequence and is present in the virion as a dimer. HE is present in a subset of beta coronaviruses and binds sialic acids on surface glycoproteins [7]. Currently, no specific therapies for SARS-CoV-2 are available and investigations regarding the treatment of SARS-CoV-2 are lacking [8]. The viral genome also encodes nonstructural proteins which can be potential drug targets, including RNA-dependent RNA polymerase (RdRp), CoV main protease (3CLpro), and papain-like protease (PLpro) [9, 10].

**Fig. 1:**
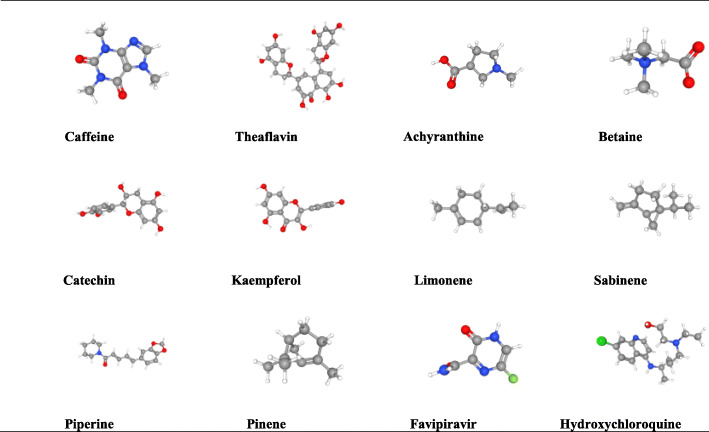
3D chemical structure of selected ligands (caffeine, theaflavin, achyranthine, betaine, catechin, kaempferol, limonene, sabinene, piperine, pinene, favipiravir, hydroxychloroquine)

**Fig. 2:**
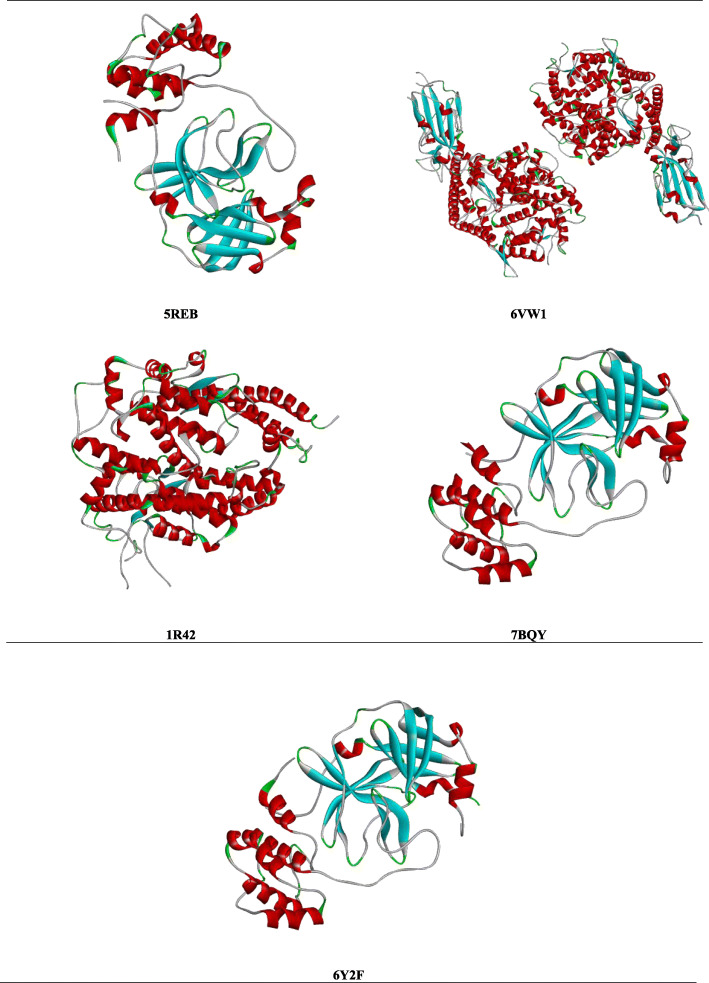
3D structure of the pure receptors (5REB, 6VW1, 1R42, 7BQY, and 6Y2F) of SAR-CoV-2

SARS-CoV-2 (2019-nCoV) outbreak has become a global pandemic that has raised the concern of the scientific community to design and discover a definitive cure for this deadly virus. Research institutions are accelerating the discovery of vaccines and therapies for the SARS-CoV-2. During the epidemic and pandemic outbreak of new viral pathogens, the conventional method of developing drugs and vaccination is not possible to control as it is a time-consuming process [11]. Consequently, the rapid approach based on in silico informatics has become very popular with recent advances in sequencing many pathogen genomes and protein sequence databases [12]. The steady rise of corona patient’s with high mortality rate reinforces the urgency to produce a safe and efficient vaccine. The pharmacological effect of phytochemicals can be adequately explained by the use of virtual screening [13]. Methods and resources like computer-aided drug discovery (CADD) is also an effective way to design new pharmaceutical products. An effective molecular interaction through molecular docking can activate the native ligand to detect the three-dimensional binding site of the enzyme and link to the relationship to the accompanying chemical compounds [14]. Taking the truth into account, a molecular docking study has been conducted to explain the phytoconstituents binding affinity with the five SARS-CoV-2 receptors. In this work, pass prediction, molecular docking, and pharmacokinetic regarding Lipinski’s rules were checked for twelve ligands. To investigate the binding affinity of the ligands and SARS-CoV-2’s receptors, the ligands were docked with the main protease and human angiotensin-converting enzyme 2 (ACE2) receptors of the SARS-CoV-2 virus owing to the best-characterized target among the SARS-CoV-2. Mainly, the binding interaction of caffeine, theaflavin, achyranthine, betaine, catechin, kaempferol, limonene, sabinene, piperine, pinene, favipiravir, and hydroxychloroquine (Fig. 1) against the proteins of COVID-19 have been screened by implying molecular docking simulations. The main protease (Mpro) (PDB ID: 7BQY, 6Y2F, 5REB) and human angiotensin-converting enzyme 2 (PDB ID: 6VW1, 1R42) were used as the target of the ligand interaction (Fig. 2). It was also previously reported that polyphenols, flavonoids, tannin, and derivate have fighting endeavors against many virions [15–17]. These components were chosen based on their therapeutic properties to bind with the SARS-CoV-2 proteins. Again, the mechanism of action of the established antiviral compounds like favipiravir and hydroxychloroquine is not fully uncovered. These compounds have the probable capacity to bind with the pockets of SARS-CoV-2; the study was encouraged to investigate the potential binding affinity of selected proteins with the previously selected ligands.

## Methods

### Bioactive constituents from black tea (*Camellia sinensis* L.)

Tea (*Camellia sinensis* L.) is the second most used non-alcoholic beverage in the world after water. It has been consumed traditionally for about 5000 years. Black tea has 3 to 6% thearubigin, theaflavin, phenolics, and catechin. Thearubigins of tea are responsible for the deep red color of the tea, while theaflavins are accountable for the astringency and red/orange color of the tea. In black tea, the highest number of phenolic pigments in black tea is thearubigin. Theaflavins individually claimed to show antiviral potency [18]. Caffeine is one of the most popular natural stimulants which can be found in tea, cacao, and coffee [19]. A cup of black tea may contain 47 to 90 mg of caffeine [20]. Caffeine seems to have a strong behavioral effect which is widely used as CNS stimulants. The phenolic component of tea was reported to have antiviral efficacy over adenovirus [21, 22].

### Bioactive constituents from prickly chaff (*Achyranthes aspera* L.)

The herb is well known owing to have anti-inflammatory, antidot (snake and scorpion bite), carminative, gastric, blood purifier, and diuretic effects. Decoction of the entire plant has been used in ascites, eczema, gastritis, skin rashes, and boils, and juice of the leaves often used to treat toothache [23]. The constituents of this pant are also reported to have antiviral properties [24, 25]. Though the entire plant ash has been used in herbal medicines for asthma, gaseous distention, urinary bladder stones, gastritis, and cough, honey ash can also be used to alleviate cough [26]. Achyranthine and betaine are abundantly found in *A. aspera* [27]. Betaine (BET) is known to protect the liver from toxicants as an indigenous drug. Betaine inhibits hepatitis B viruses with the advantages of reducing resistance to interferon and lamivudine [28].

### Bioactive constituents from catechu (*Areca catechu* Linn.)

Areca nut (*Areca catechu*) is frequently used in betel quid, often consumed in betel and lime. Betel quid chewing seems to be extremely common, particularly in many countries in Southeast Asia [19]. *Areca catechu* is one of 54 recognized alkaloid-containing species in the Areca [29]. Several active ingredients such as arecatannin B1, procyanidins, and seed extracts have shown HIV enzyme inhibitory function. Alkaloids (0.5 %), phenol (31.1%), fiber (10.8%), fat (14.0%), and polysaccharide (18.7%) are key elements of the fruits of *Areca catechu* L. Alkaloids and polyphenols are the primary components in flower [30]. In *Areca catechu*, catechin and kaempferol were identified previously [31].

### Bioactive constituents from lemon (*Citrus limon*)

*Citrus limon* is a flowing plant in the Rutaceae family [32]. The compounds of Citrus plants are the most common essential oil sources used in food products and medicines. Lemon seems to be very abundant in natural substances such as flavonoids, citric acid, minerals, ascorbic acid, and essential oils. Although modern citrus cultivars were primarily designed for fresh use, their naturally drawn constituents, such as the flavonoids and phenolic compound, contributed to their use throughout food technology and pharmacological research [33]. Valuable academic publications concentrate on lemon fruit extract, juice, and essential oil, which are becoming much more comprehensive in pharmacological terms. These reports claim antiviral, antibacterial, anti-cancer, anti-inflammatory, and cardioprotective activities of lemon [34]. Sabinene (15.9%) and limonene (31.5%), are the major constituents of the citrus lemon [35]. Citrus flavonoids exert several pharmacological potentialities, including antiviral action [33].

### Bioactive constituents from black pepper (*Piper nigrum*)

Among the kitchen spices, pepper is one of the most commonly used spices around the globe. Pepper contains piperine and due to the pungent odor of the piperine, it has taken place as a kitchen spice. It is commonly used and widely permissible in various western medicinal systems, such as Unani and Ayurvedic [36]. Many illnesses, including antihypertensives [37, 38], antiplatelets, antioxidant, analgesic, antitumor, antithyroid, anticonvulsant [39], antibacterial, antidiarrheal, antidepressants, antispasmodic, immunomodulatory, anti-inflammatory, hepatoprotective, and antifungal have already been treated by this plant [37]. As a result, studies on its derivative synthesis, SAR modification and biological tests are underway and this has encouraged researchers to keep investigating it [40]. Piperine is also used in both conventional Chinese and Indian therapies. In influenza, nausea, rheumatoid arthritis, fever, and chills, piperine is used extensively [41]. Again, the previous studies reported that pinene has a strong suppression capacity for the herpes simplex virus [42]. It has been used for centuries to manufacture aromas and fragrances and demonstrates fungicidal activity. Various pharmacological activities, including natural insecticide, are attributed to pinenes. The studies showed strong antiviral potency of pinens against infectious bronchitis virus [42].

### Synthetic bioactive compounds (favipiravir and hydroxychloroquine)

Favipiravir and hydroxychloroquine are under examination for the treatment of SARS-CoV-2, but the antiviral efficacy of these drugs is not apparent [43]. Favipiravir is a nucleoside precursor approved for the treatment of pandemic influenza in Japan [44]. However, its antiviral effect in patients with COVID-19 needs demanding data to support [45]. Hydroxychloroquine is a safer analog of chloroquine that has fewer concerns about drug-drug interactions [46]. Over one research, patients with combined treatment had lower viral loads in contrast to patients with a similar viral load-receiving hydroxychloroquine alone [47]. The suppression of cytokine release has been accelerated by downregulating the inflammatory effect of the drug [48]. Inhibition of cytokines reduces the elevation of chemotaxin of polymorphonuclear leucocytes in the lungs, which finally decreases the orientation of reactive oxygen [49].

### Protein targets

Several approaches have been taken to establish CoV vaccines, most of which aim against spikes (S) or S-proteins, as they are the primary adjuvant to collapse antibodies. The main protease (M-pro) enzyme has been described as one of the primary goals for producing antiviral vaccines or drugs [50]. M-pro is present in the SARS-CoV-2’s polyprotein ORF1ab and is important for virus replication. This protease participates in the degeneration of polyprotein [51]. Except for a residue (Ala285Thr), the association of the M-pro enzyme has been highly identified in the SARS-CoV virus [52]. In addition, two subunits S1 and S2, are found in the S protein molecule. Again, the ACE-2 receptor is linked to the S2 subunit and within this receptor, the S2 subunit forms fusion between the membrane of the host cell and the virus. As a result, the viral RNA can access the cytoplasm of the viral cell and replicate again [53]. Vaccine regarding S protein can also trigger antibodies that obstruct the binds of a viral receptor and even the uncoating of the viral genome in the cytoplasm. The S protein-based vaccine will play a major role in inducing protective immunity from SARS-CoV infection by neutralizing the T cell responses and triggering antibodies [53]. Based on the full-length genome phylogenetic study, SARS-CoV-2 has an almost 89% similarity with the SARS-CoV [54]. This was the foundation for the initial development of SARS-CoV-2 and indicated that the receptor of SARS-CoV-2 may be consistent with the receptor of SARS-CoV (ACE2) [55]. SARS-CoV-2 is reported to use angiotensin-converting enzyme 2 (ACE2) receptors to penetrate the target cells [56]. Therefore, any agent increasing ACE2 production may be anticipated to improve its susceptibility to intense COVID-19 by enhancing viral cellular invasion. However, biochemically, Angiotensin II is converted to angiotensin (1–7), defending lung damage by reducing the ACE2 receptor and vasodilation [57]. The patients with COVID-19 and co-morbidities such as hypertension, cardiovascular disorder, and diabetes that often have angiotensin-converting enzyme inhibitors ACEIs or angiotensin receptor blockers ARBs, there is often contradictory evidence about continuity or discontinuation of medications inhibiting the renin-angiotensin aldosterone system (RAAS), including inhibitors of angiotensin-converting enzyme (ACEIs) and angiotensin receptor blockers (ARBs) [58]. 5REB [59], 7BQY [60], and 6Y2F [61] have been previously denoted as main protease (Mpro) of SARS-CoV-2, and 6VW1 [62] and 1R42 [63] have been denoted as human angiotensin-converting enzyme 2 (ACE2). Taking all these considerations into account, five proteins (5REB, 7BQY, 6Y2F, 6VW1, 1R42) have been selected to comply with the binding interactions between proteins and ligands.

### Pass prediction

Caffeine, theaflavin, achyranthine, betaine, catechin, kaempferol, limonene, sabinene, piperine, pinene, favipiravir, and hydroxychloroquine were allowed to predict the antiviral activity by using the PASS online server [64]. PASS online server predicts the activity as probable of activity (Pa) and probable inactivity (Pi).

### Molecular analysis: compounds and target proteins

Three-dimensional structures of the main protease in complex (hydrolase receptor) (PDB: 5REB) [65], the structure of SARS-CoV-2 chimeric receptor-binding domain complexed with its human receptor ACE2 (PDB: 6VW1) [66], native human angiotensin-converting enzyme-related carboxypeptidase (ACE2) (PDB: 1R42) [67], the crystal structure of the COVID-19 main protease (PDB: 7BQY) [68], and crystal structure (monoclinic form) of the COVID-19 main protease were downloaded in PDB format. Besides, the chemical compounds have been selected based on the bioactive dietary compounds and synthetic compounds (caffeine, theaflavin, achyranthine, betaine, catechin, kaempferol, limonene, sabinene, piperine, pinene, favipiravir, and hydroxychloroquine) including antiviral properties. The compounds have been derived from the PubChem database (https://pubchem.ncbi.nlm.nih.gov/) [69] in SDF format.

### Molecular analysis: preparation of target protein and compounds

The ligands have been imported in 2DSDF format; hereafter, the ligands have been minimized and converted to pdbqt format across the PyRx tools to find the best hit in these targets. The protein structure was created with Discovery Studio and UCSF Chimera. Default settings in the PyRx from the MGL tools [70] have been used for the virtual screening. Through the Discovery Studio 2020, all water and the heteroatom were eliminated from proteins. A combination of non-polar hydrogen and the Gasteiger charge was used to assemble proteins. Besides, extra solvents were deleted, selenomethionine (MSE), methionine (MET), bromo UMP to UMP (U), methylselenyl-dUMP (UMS), to UMP (U), methylselenyl-dCMP (CSL) to CMP (C) have been marked to keep only highest occupancy. Again, the incomplete side chains were replaced by Dunbrack 2010 rotomer library. Furthermore, all proteins have been lowered to the least energy level by keeping the residues in AMBER ff14sB and Gasteiger mode in UCSF Chimera [71].

### Molecular analysis: molecular docking

For the protein-ligand binding operation of the selected protein-ligand complexes, PyRx Autodock Vina was used [26]. A semi-flexible docking system has been applied to perform the docking research. A semi-flexible docking system has been applied to perform the docking research. The phytochemicals were translated into PDBQT formats with PyRx AutoDock tools. The rigidity of proteins and ligands was retained for this analysis. Ligand molecules had given the freedom for 10 degrees. AutoDock determines the molecules to format pdbqt, box style, grid box formation, etc. The grid box with an active position was built in the middle of the box. BIOVIA Discovery Studio Visualizer 2020 [72] was eventually accelerated to evaluate the docking sites for the possible linking approaches.

### Determination of pharmacokinetic parameters by Swiss ADME

Lipinski’s rule evaluates the different descriptors which are important for drug design. According to Lipinski’s rule, an orally active drug should fulfill the following drug-likeness parameters to demonstrate their pharmaceutical fidelity such as (i) molecular mass less than 500 Daltons, (ii) no more than 5 H-bond donors, (iii) no more than 10 H-bond acceptors, and (iv) O/W partition coefficient log P not greater than 5. If the molecule violates more than 3 descriptor parameters, it will not fit into the criteria of drug likeliness, and it is not considered to proceed with drug discovery.

## Results

### Pass prediction of selected ligands

The results of the PASS prediction have been presented in Table 1. The study revealed that, among the four compounds, favipiravir and theaflavin retained the highest drug probability Pa (0.662 and 0.609).

**Table 1:** Pass prediction of caesalpinine A, diffutidin, favipiravir, hydroxychloroquine antiviral activity

Chemical constituents												
Antiviral activity	Caffeine	Theaflavin	Achyranthine	Betaine	Catechin	Kaempferol	Limonene	Sabinene	Piperine	Pinene	Favipiravir	Hydroxychloroquine
Pa	0.513	0.609	0.590	0.525	0.520	0.496	0.574	0.282	0.221	0.346	0.662	0.323
Pi	0.045	0.012	0.021	0.040	0.018	0.005	0.009	0.105	0.167	0.066	0.008	0.207

*Pa* probability of activity, *Pi* probability of inactivity

### Molecular docking study for SARS-CoV-2 inhibition

**Fig. 3:**
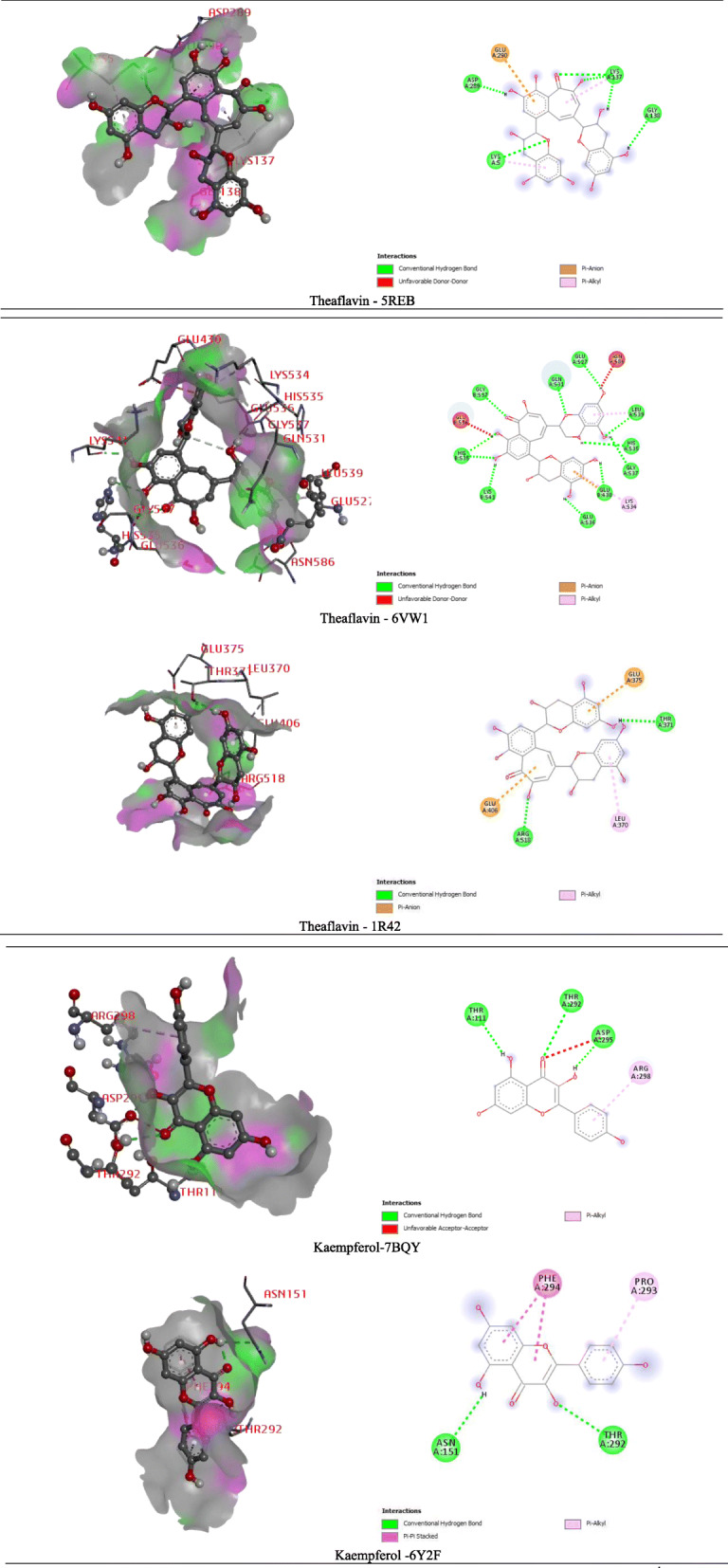
The presentation of the best binding interactions of the selected bioactive dietary components and synthetic compounds with the five SARS-CoV-2 proteins (main protease and angiotensin-converting enzyme 2 receptors)

In the case of the antiviral docking study, the selected compounds were docked against the SARS-CoV-2’s main protease (PDB: 5REB, 7BQY, 6Y2F), and angiotensin-converting enzyme 2 (PDB: 6VW1, 1R42) enzymes and displayed docking scores ranging from −3.2 to −9.8 (kcal/mol). The docking score and the best interaction figure have been shown in Table 2 and Fig. 3. From the findings, it was observed that the compound theaflavin exposed the highest score against PDB ID: 5REB, 6VW1, and 1R42, followed by kaempferol attained the highest binding affinity with the pockets of the main protease enzyme (Mpro) (7BQY and 6Y2F). The ranking of docking score for antiviral (SARS-CoV-2) effect against PDB ID: 5REB is as follows: theaflavin > kaempferol > favipiravir > hydroxychloroquine > caffeine > limonene > sabinene > achyranthine > betaine. Docking observed docking score and with the selected components and PDB ID: 6VW1 receptor, the scores were observed: theaflavin (−9.8 kcal/mol), kaempferol (7.0 kcal/mol), piperine (−6.8 kcal/mol), catechin (−6.7 kcal/mol), hydroxychloroquine (5.4 kcal/mol), limonene (4.8 kcal/mol), caffeine (4.8 kcal/mol), favipiravir (−4.7 kcal/mol), sabinene (4.5 kcal/mol), pinene (4.5 kcal/mol), achyranthine (4.1 kcal/mol), and betaine (3.2 kcal/mol) and respectively. Theaflavin yields the highest docking score against the 1R42 receptor. Theaflavin binds to the pocket of the 1R42 receptor through a series of bonds: conventional hydrogen bond (arg518, thr371), Pi-anion (glu406, glu375), and Pi-alkyl (leu370). The order of docking score against PDB ID: 7BQY is as follows: kaempferol > catechin > theaflavin > piperine > hydroxychloroquine > favipiravir > limonene > pinene > achyranthine > sabinene > betaine. Caffeine does not conjugate and neither oriented any docking to this ACE2 receptor. Again, kaempferol also exerted the best docking score as well as the best binding affinity to the main protease enzyme (PDB: 6Y2F). Kaempferol obtained the highest binding score via interaction of the amino acid series (asn158, phe294, pro293, and thr292) of the protein.

**Table 2:** Docking score of the selected dietary bioactive and synthetic compounds

Compounds	Docking score				
	5REB	6VW1	1R42	7BQY	6Y2F
Caffeine	−5.1	−4.8	−5.7	-	−5.0
Theaflavin	−**6.3**	−**9.8**	−**8.6**	−6.7	−6.4
Achyranthine	−4.4	−4.1	−4.0	−4.2	−4.7
Betaine	−3.5	−3.2	−3.2	−3.4	−3.5
Catechin	-	−6.7	−6.9	−7.0	−6.5
Kaempferol	−6.2	−7.0	−6.8	−**7.1**	−**6.6**
Limonene	−4.6	−4.8	−4.7	−4.4	−5.2
Sabinene	−4.5	−4.5	−4.8	−4.1	−4.7
Piperine	-	−6.8	−7.0	−6.5	−7.1
Pinene	-	−4.5	−4.5	−4.4	−4.6
Favipiravir	−5.4	−4.7	−5.2	−5.5	−5.3
Hydroxychloroquine	−5.4	−5.6	−5.8	−5.7	−6.0

### Pharmacokinetics (ADME) and toxicological properties prediction

The study was performed to check the pharmacokinetics and the toxicological properties of the caffeine, achyranthine, betaine, catechin, kaempferol, limonene, sabinene, and sabinene, piperine, pinene, favipiravir, and hydroxychloroquine. The pharmacokinetics and the toxicological properties of the selected ligands for the antiviral (SAR-CoV-SAR) efficacy have been shown in Table 3. The study revealed no violation of Lipinski’s five rules and the ligands does not contain carcinogens.

**Table 3:** Absorption, distribution, metabolism, excretion, and toxicological properties of the selected compounds

Compounds	Molecular Weight (M.W) (g/mol)	HBD	HBA	log P (o/w)	HIA	Carcinogenicity (binary)	Violation score
Caffeine	194.19	0	6	−1.03	0.9824	0.9429	0
Theaflavin	564.50	12	9	2.21	0.9836	0.9571	3
Achyranthine	129.16	1	2	0.02	0.9021	0.9571	0
Betaine	117.15	0	2	−1.56	0.9333	0.7316	0
Catechin	290.27	5	6	1.55	0.9887	0.9286	0
Kaempferol	286.24	4	6	2.28	0.9881	0.6985	0
Limonene	136.24	0	0	3.31	0.9692	0.5856	0
Sabinene	136.24	0	0	3.00	0.9828	0.7286	0
Piperine	285.34	0	3	3.00	0.9639	0.9198	0
Pinene	136.24	0	0	3.14	0.9677	0.7286	0
Favipiravir	157.10	2	3	−0.99	0.9612	0.9286	0
Hydroxychloroquine	335.88	2	4	3.78	0.9934	0.8429	0

*HBD* hydrogen bond donor, *HBA* hydrogen bond acceptor, *LogP* lipophilicity, *HIA* human intestinal absorption

## Discussion

Molecular docking and computer-aided drug design (CADD) are vital tools in structural molecular biology. This tool contributes to predicting the binding mode of active compounds against the targeted proteins [73]. Additionally, it is used to comprehend the possible molecular mechanism of actions of various pharmacological activities [74]. The molecular docking was also performed to correlate with our current research outcomes and better understand the molecular mechanism [72]. In this study, twelve bioactive and synthetic compounds were examined against five targeted receptors or enzymes. The ideal antiviral drug candidate should have low toxicity, appropriate pharmacokinetics, and preferably a large spectrum of activity. Many adjuvants failed to be clinically used due to the lack of one or more of these properties [75]. In this study, 10 bioactive dietary constituents have been selected owing to their wide availability with antiviral efficacy. Besides, favipiravir and hydroxychloroquine have been pronounced to antiviral efficacy in previous investigations, though the investigations were not successfully proven [76–79]. The search for effective inhibitors of 5REB, 6Y2F, 6VW1, 1R42, and 7BQY has been the focus of drug discovery efforts and has led to the identification of some promising leads (caffeine, theaflavin, achyranthine, betaine, catechin, kaempferol, limonene, sabinene, piperine, pinene, favipiravir, and hydroxychloroquine) have shown good inhibitory activity on selective enzymes. Theaflavin yielded highest binding score among all the selected compounds. It scored −6.3, −9.8, and −8.6 (kcal/mol) upon it was docked with the 5REB, 6VW1, and 1R42 enzymes. Theaflavin binds to the pocket of 6VW1 receptor through a series of bonds: conventional hydrogen bond (lys541, his535, gly537, gln531, glu527, leu539, gly537, glu430, gly536), Pi-alkyl (lys534), unfavorable donor (glu536, asn526), and resulted best binding affinity among the selected compounds. Besides, kaemperol showed highest binding affinity to the rest of the receptors (Fig. 3 and Table 2).

Favipiravir is one of the antiviral drugs tested against several SARS-CoV-2 envelope proteins [80]. Favipiravir, a pyrazine carboxamide derivative molecule, works as an antiviral molecule that is a prodrug against RNA viruses. This antiviral activity is attenuated by the competitive nature of favipiravir with purine nucleoside rather than pyrimidine nucleosides. Madin Darby Canine Kidney (MDCK) cell treated with favipiravir during in vitro assay provided favipiravir ribofuranosyl-5′-triphosphate (favipiravir-RTP), favipiravir ribofuranose (favipiravir-R), and favipiravir ribofuranosyl-5′-monophosphate (favipiravir-RMP) in HPLC analysis as metabolites among which chemically synthesized favipiravir-RTP showed positive outcome to inhibit the viral RNA polymerase activity in concentrations ranging from nanomolar to micromolar. This assay infers the projection that favipiravir, a prodrug, emits the antiviral property when it is intra-cellularly phosphoribosylated to be an active form, favipiravir-RTP to halt RNA polymerase. When a single molecule of favipiravir-RTP is efficiently incorporated into a nascent RNA strand, a partial inhibition of extension in viral RNA is observed and when double incorporation takes place, complete inhibition results in [81].

Despite not being an elucidated absolute mechanism of action of hydroxychloroquine against coronavirus, several mechanisms to elucidate its antiviral property are already proposed. Being a weekly basic drug, hydroxychloroquine upraises the pH within cells and at the cellular membrane, consequently inhibiting the virus’s ability to fuse onto the cell membrane and enter host cells. Another proposed hydroxychloroquine mechanism includes inhibition of DNA and RNA synthesis along with immunomodulating and anti-inflammatory effects [82]. Hydroxychloroquine also infers the constraint of glycosylation of viral proteins, virus assembly, new virus particle transport, virus release, and other processes to achieve its antiviral effects [83]. Docking studies indicated that the binding interaction of selected bioactive and synthetic constituents with 5REB, 6Y2F, 6VW1, 1R42, and 7BQY had been concluded that the selected constituents might, in part, be responsible for the antiviral activity against SARS-CoV-2 through interactions with these target enzyme or receptor.

## Conclusions

Theaflavin and kaempferol exert a high binding affinity to the pockets of SARS-CoV-2 proteins. Interaction of caffeine, theaflavin, achyranthine, betaine, catechin, kaempferol, limonene, sabinene, piperine, pinene, favipiravir, and hydroxychloroquine with main protease and angiotensin-converting enzyme 2 (ACE-2) proteins is ensured by molecular docking, which accredits the assertion of remarkable antiviral properties of these test moieties. Owing to design drugs for anti-COVID-19, this is just a primary investigation and further investigations are suggested to confirm the capacity of the ligands.

## Data Availability

Data and material are available upon request.
